# The cost-effectiveness of vitamin D supplementation for the prevention of type 2 diabetes in the United States: a microsimulation modelling study

**DOI:** 10.1016/j.lana.2026.101543

**Published:** 2026-06-27

**Authors:** Jonathan Briody, Anastassios G. Pittas, Ping Zhang, Yixue Shao, Edward Gregg

**Affiliations:** a Converge: Centre for Chronic Disease and Population Health Research, School of Population Health, RCSI University of Medicine and Health Sciences, Dublin, Ireland; b Division of Endocrinology, Diabetes and Metabolism, Department of Medicine, Tufts Medical Center, Boston, MA 02111, USA; c Division of Diabetes Translation, Centers for Disease Control and Prevention, Atlanta, GA, USA

**Keywords:** Vitamin D, Type 2 diabetes, Cost-effectiveness, Microsimulation, Prediabetes, Prevention

## Abstract

**Background:**

Vitamin D lowers the risk of type 2 diabetes in adults with prediabetes. We assessed the long-term cost-effectiveness of vitamin D supplementation for preventing type 2 diabetes.

**Methods:**

We applied a validated microsimulation model to estimate long-term health and economic consequences of vitamin D in a representative cohort of 10,000 U.S. adults with prediabetes in the National Health and Nutrition Examination Survey (NHANES) 2013–2018 cohort. Treatment effects came from individual-participant data of three randomised trials of vitamin D versus placebo (D2d, USA, 2013–2018; DPVD, Japan, 2013–2019; Tromsø, Norway, 2008–2015; pooled analysis 2023). Cost-effectiveness was measured by an incremental cost-effectiveness ratio (ICER) in terms of cost per quality-adjusted life year (QALY) compared to no vitamin D. We conducted subgroup analyses by age, baseline fasting glucose level, body weight, and family history of diabetes. We performed one-way and probabilistic sensitivity analysis. Costs were in 2024 U.S. dollars. 10-year and lifetime time horizons were studied from a U.S. health care system perspective.

**Findings:**

In a NHANES-derived cohort of 4176 adults with prediabetes (mean age 53.3 years [SD 17.0]; 2024 [49%] female; 2603 [62%] non-Hispanic White, 488 [12%] non-Hispanic Black, 668 [16%] Hispanic, 417 [10%] other race/ethnicity), vitamin D supplementation reduced per-person lifetime costs by $3208 (95% uncertainty interval [UI] −$5974 to $171), increased lifetime QALYs by 0.12 (95% UI 0.05–0.19), and yielded a lifetime net monetary benefit of $15,483 (95% UI $8108–$21,907) at a willingness-to-pay threshold of $100,000/QALY. Life-years gained were 0.27 per person (95% UI 0.06–0.50). Vitamin D reduced the lifetime cumulative incidence of diabetes by 8% (95% UI 6.4–10.6%). Diabetes complications were reduced by an average of 6.4% (95% UI 3.2–8.7%). There were little differences in ICERs across subgroups. Changes in intervention-related parameters had limited effects on the cost-effectiveness ratios across sensitivity analyses, and the intervention likewise remained cost-saving compared to no vitamin D under all simulation scenarios.

**Interpretation:**

Vitamin D supplementation may be an efficient use of health care resources for preventing type 2 diabetes in adults with prediabetes.

**Funding:**

Research Ireland (Grant Number: EPSPD/2022/165).

**Research in context**

**Evidence before this study**

We searched PubMed, Embase, and Cochrane CENTRAL from database inception to March 1, 2026, for studies evaluating vitamin D supplementation for prevention of type 2 diabetes in adults with prediabetes. Search terms included “vitamin D”, “cholecalciferol”, “25-hydroxyvitamin D”, “prediabetes”, “type 2 diabetes”, “diabetes prevention”, “cost-effectiveness”, and “economic evaluation”. We also reviewed references from retrieved articles, meta-analyses, and clinical practice guidelines. Three randomized, placebo-controlled trials in adults with prediabetes–D2d (USA), DPVD (Japan), and the Tromso vitamin D and T2DM trial (Norway)–showed modest, non-statistically significant reductions in the incidence of diabetes with vitamin D supplementation. An individual participant data meta-analysis of these trials showed a significant 15% reduction in the risk of diabetes and a higher likelihood of regression to normoglycemia with vitamin D supplementation. These findings informed the 2024 Endocrine Society Clinical Practice Guideline on Vitamin D for the Prevention of Disease, which recommended vitamin D supplementation for adults with high-risk prediabetes. Despite growing evidence of benefit, evidence on whether vitamin D supplementation is a cost-effective or cost-saving strategy for preventing type 2 diabetes is limited.

**Added value of this study**

This study evaluates the cost-effectiveness of vitamin D supplementation for diabetes prevention integrating randomized trials and meta-analyses. The findings show that the modest but consistent reduction in diabetes risk with vitamin D supplementation may translate into meaningful long-term health and economic benefits. The analysis shows that vitamin D supplementation for diabetes prevention is not only cost-effective but potentially cost-saving. Importantly, the findings indicate that greater economic benefits may accrue to individuals at higher baseline risk of diabetes progression (e.g., higher BMI, higher fasting glucose, younger age, and family history of diabetes), providing actionable insights for targeted implementation strategies. This work addresses a knowledge gap identified by the Endocrine Society guidelines, showing that savings from the avoidance or delay of diabetes and its associated complications more than offset the low cost of the supplement itself–providing evidence that vitamin D is an economically viable strategy for diabetes prevention at scale.

**Implications of all the available evidence**

Taken together, the available evidence suggests that vitamin D supplementation is a safe, low-cost intervention that modestly reduces the progression from prediabetes to type 2 diabetes and may lower long-term healthcare costs. Vitamin D supplementation may therefore represent a practical addition to diabetes prevention strategies, particularly for high-risk populations and settings where more intensive interventions are less feasible. Given the large and growing global burden of prediabetes, even modest relative reductions in diabetes risk could translate into substantial public health benefit. The low cost, accessibility, and favorable safety profile of vitamin D supplementation may also support more equitable implementation across low-resource populations disproportionately affected by diabetes. Future research should focus on implementation strategies, long-term adherence, and identification of individuals most likely to benefit, including evaluation of baseline risk, achieved vitamin D concentrations, and genetic factors that may modify treatment response.

## Introduction

Diabetes is one of the world’s greatest public health threats, now affecting close to 15% of all U.S. adults and leading to a significant burden of morbidity and increased health care costs for individuals, health systems, and national economies.^1^^,^^2^ Worldwide, an estimated 828 million adults had diabetes in 2022.^3^ Approximately 9 in 10 individuals with diabetes have type 2 diabetes, which is often preceded by prediabetes. About 38% of the adult U.S. population has prediabetes.^1^ Globally, the prevalence of impaired glucose tolerance was 9.1% (464 million people) in 2021, with projections suggesting an increase to 10% (638 million people) by 2045.^4^ Despite the global growth in prevalence, type 2 diabetes is thought to be preventable by addressing various behavioural, environmental, and physiological factors, including diet, physical activity, air quality, and stress.^5^ Randomized controlled trials aimed at preventing type 2 diabetes have primarily focused on intensive lifestyle interventions, metformin, bariatric surgery, and weight loss medications targeting people with prediabetes.^6^ However, the costs and risks associated with these interventions have thus far had limited scalability to only small segments of those who are at high risk of type 2 diabetes.^7^^,^^8^ There is a persistent and urgent need to identify other modifiable risk factors and safe, affordable, and easily accessible interventions to prevent type 2 diabetes and alleviate the disease burden.

The role of vitamin D in skeletal health is well established, but its potential effects on extra-skeletal diseases, especially type 2 diabetes, have gained attention in the past decade. Longitudinal observational studies have reported a 28%–38% diabetes risk reduction comparing people at the highest to lowest 25-hydroxyvitamin D or 25(OH)D, levels.^9^, ^10^, ^11^ Vitamin D may influence glucose metabolism by stimulating insulin secretion through receptors on pancreatic beta cells, reducing systemic inflammation, and enhancing insulin sensitivity.^12^

Three trials designed to test whether vitamin D reduces the rate of progression to diabetes in people with prediabetes found consistent reductions in diabetes risk compared to placebo.^13^, ^14^, ^15^ The differences were not statistically significant because the observed risk reductions (10%–13%) in the individual trials were smaller than each trial was powered to detect (25%–30%). A meta-analysis of individual participant data from these three trials showed a statistically significant 15% reduction in the risk of progression from prediabetes to diabetes with vitamin D supplementation.^2^ Other meta-analyses that combined aggregate data from these three trials and smaller, short-term trials found similar reductions in diabetes risk with vitamin D in adults with prediabetes.^16^, ^17^, ^18^ Based on this evidence of a modest yet consistent benefit of vitamin D in preventing type 2 diabetes, the 2024 Endocrine Society Guideline on Vitamin D for the Prevention of Disease includes a conditional (GRADE 2) recommendation for vitamin D supplementation for diabetes prevention, reflecting moderate certainty of evidence (+++O) and an assessment that benefits likely outweigh risk.^19^ The Guideline notes some limitations, including the allowance of background vitamin D supplementation in some studies, which may have attenuated observed treatment effects. Although the preventive effect of vitamin D supplementation at the individual level appears modest, the direction and magnitude of effect are consistent across trials. When applied at scale, even small risk reductions from a low-cost intervention could translate into meaningful population-level benefits for diabetes prevention, particularly in low-resource settings.

The long-term cost-effectiveness of vitamin D supplementation for preventing type 2 diabetes warrants evaluation. As health benefits and medical costs saved from preventing type 2 diabetes and its complications associated with vitamin D supplementation will accumulate over time and long-term follow-up of vitamin D clinical trials is unlikely due to costs, a computer simulation may serve as a means to extrapolate these long-term health and economic benefits. In this study, we assessed the long-term cost-effectiveness of vitamin D supplementation for preventing type 2 diabetes in the U.S. adult population with prediabetes.

## Methods

Using the model described below, we simulated the lifetime costs and quality-adjusted life years (QALYs) of vitamin D supplementation in U.S. adults aged 18 years with prediabetes compared with no intervention. We used incremental cost-effectiveness ratios (ICERs), which are calculated as differences in costs in a monetary term divided by the difference in effectiveness in QALYs with and without the intervention, to measure the cost-effectiveness. We adopted a health system perspective that considered direct medical costs, expressed in U.S. dollars (year 2024). The annual discount factor for future costs and QALYs was 3%. We also calculated net monetary benefit (NMB) to rank the results across subgroups as ICERs could be negative. Using the U.S. willingness-to-pay (WTP) threshold of $100,000 per QALY,^20^ NMB is calculated as NMB = (QALYs gained × WTP) − cost. An intervention is deemed cost-effective when the NMB is greater than zero, indicating that health benefits outweigh costs relative to the WTP.

### Overview of the simulation model

We used the type 2 diabetes simulation model developed by the Centers for Disease Control and Prevention (CDC) and Research Triangle Institute (RTI) International.^21^ The model was designed to project the long-term cost-effectiveness of lifestyle interventions and medication therapy to prevent type 2 diabetes among people at high risk for the development of diabetes and for interventions to manage risks for diabetes-related complications among people with type 2 diabetes. The model is a discrete-time microsimulation with annual steps simulating individual risk, complications, and mortality until death to calculate costs, QALYs, and ICERs of interventions relative to a baseline by incorporating time-varying risk factors and disease history. The prediabetes module tracked costs and QALYs for individuals who have not yet developed type 2 diabetes, including those with cardiovascular complications and mortality before developing diabetes. The diabetes module model tracked the development of diabetes complications and death for people who developed diabetes. The diabetes module contained a set of risk equations for 17 diabetes-related complications and mortality. The model allows some complications to occur more than once during the simulation. Progression to diabetes complications is as described by prior research.^21^ The model has been validated against the results of many major clinical trials and cohort studies and found to accurately predict the development and progression to diabetes and its complications.^21^

### Study population

A nationally representative sample of U.S. adults (aged≥18 years) with prediabetes was generated by using the 2013–2018 National Health and Nutrition Examination Survey (NHANES), which included information on baseline vitamin D. Prediabetes was defined as those individuals having an HbA1c value between 5.7% and 6.4% (inclusive), or a fasting plasma glucose (FPG) value between 100 and 125 mg/dL (inclusive), or 2-h glucose value after a 75-g oral glucose challenge between 140 and 199 mg/dL. People with self-reported diagnoses or other diagnostic criteria of diabetes (e.g., HbA1c ≥ 6.5% or FPG ≥ 126 mg/dL) excluded. The characteristics of this sample including demographic variables, risk factors, and previous history of diabetes-specific complications (Table 1) were applied to generate a simulated population of 10,000 individuals for the simulation. Race and ethnicity were self-reported by NHANES participants using pre-specified categories: non-Hispanic White, non-Hispanic Black, Hispanic, and other race/ethnicity (including non-Hispanic Asian, multiracial, and other groups). These categories are sociocultural constructs and are used here to describe the representativeness of the study population and to explore potential disparities in vitamin D supplementation and diabetes prevention.

**Table 1:** Demographic and clinical characteristics of NHANES survey sample (2013–2018) with prediabetes.

Variable	Mean (SD) or n (%)
Total sample size	4176
Demographics	
Mean Age, years	53.3 (17.0)
Female	2024 (49%)
Male	2152 (51%)
Non-hispanic white	2603 (62%)
Non-hispanic black	488 (12%)
Hispanic	668 (16%)
Other Race/Ethnicity	417 (10%)
Postsecondary education	2538 (61%)
Current smoker	738 (18%)
Clinical measurements	
HbA1c, %	5.7 (0.5)
Body mass index, kg/m^2^	30.3 (7.3)
Systolic blood pressure, mm Hg	128 (19)
HDL cholesterol, mmol/L	1.35 (0.39)
LDL cholesterol, mmol/L	3.00 (0.93)
Triglycerides, mmol/L	1.12 (0.67)
Serum creatinine, μmol/L	80 (35)
Fasting plasma glucose, mmol/L	5.9 (0.8)
Clinical history	
Serum 25-hydroxyvitamin D, <30 nmol/L	319 (7.6%)
Serum 25-hydroxyvitamin D, 30–50 nmol/L	913 (21.9%)
Serum 25-hydroxyvitamin D, ≥50 nmol/L	2944 (70.5%)
Microalbuminuria	341 (8.2%)
Macroalbuminuria	35 (0.8%)
CKD stage 3–5: eGFR < 60 mL/min/1.73 m^2^	405 (9.7%)
CKD stage 4–5: eGFR < 30 mL/min/1.73 m^2^	19 (0.5%)
Undergoing dialysis	2 (<0.1%)
Neuropathy	366 (8.8%)
Myocardial infarction	104 (2.5%)
Stroke	129 (3.1%)
Congestive heart failure	56 (1.3%)
Angina	116 (2.8%)
Family history of diabetes	1136 (27%)

Values are mean (standard deviation) for continuous variables and n (%) for categorical variables, calculated from the NHANES 2013–2018 sample of US adults (aged ≥18 years) with prediabetes (N = 4176) that was used to parameterise the simulated cohort. Microalbuminuria is defined as an albumin creatinine ratio >3 mg/mmol but <30 mg/mmol, or albumin concentration >20 mg/L but <200 mg/L. Macroalbuminuria is defined as an albumin creatinine ratio >30 mg/mmol or albumin concentration >200 mg/L.

### Intervention

A daily dose of 100 mcg (4000 IU) of vitamin D_3_ (cholecalciferol) was chosen for the purpose of preventing type 2 diabetes. This dosage is practical and closely approximates the 3500 IU daily weighted average used in the trials.^2^ Individuals who are identified with prediabetes received vitamin D until the onset of type 2 diabetes. The simulation assumed the study population received vitamin D supplements in addition to their background vitamin D intake (via diet or supplements) which was consistent with the vitamin D and diabetes prevention trials. The intervention was applied to individuals meeting clinical eligibility criteria of pre-diabetes and BMI ≥ 25 kg/m^2^, consistent with the 2024 Endocrine Society Guideline and the trial populations from which efficacy estimates were derived.^2^^,^^19^ We included persons with BMI < 25 in our baseline denominator but only initiate the intervention if and when individuals pass the BMI threshold of > 25 in the course of the model’s longitudinal simulation. We assumed an adherence rate of 85% among users and that vitamin D led to a 15% risk reduction of type 2 diabetes, as observed across the vitamin D and diabetes prevention trials.^2^ On development of type 2 diabetes, vitamin D supplementation was discontinued and, consistent with trial findings, no additional clinical benefit was modelled. Consequently, no direct therapeutic benefit of vitamin D on outcomes after diagnosis is assumed; other than the downstream health and economic benefits accrued from delaying an earlier disease onset and reduced lifetime complications due to a shorter duration of exposure to the diabetic state.

### Costs

Total costs include treatment costs, complication costs, and intervention costs. Cost equations include an intercept, an age term, and cost increments that are applied with values differing if the individual has a complication in the current or a previous year. Prediabetes parameters include the base costs and time-varying age costs (change in $ per year) of normoglycemia and prediabetes. We used two cost equations to reflect the different health care payment systems for people younger and older than 65 years. Cost equations once diabetes has developed for people younger than 65 years are based on equations estimated by Yang et al.^22^ For those with diabetes who were ≥ 65 years old, we applied a Medicare cost equation estimated by Wang et al.,^23^ which is described in the Appendix (Table A1). The annual (incremental) cost of the intervention and costs associated with diabetes and disease-related complications are included as key parameters for the model, as described in Table 2. All costs were adjusted to 2024 U.S. dollars using the GDP price index from the U.S. Bureau of Economic Analysis.^25^ Supplementation cost was calculated from the average retail cost of a 30-day supply of vitamin D_3_ 4000 IU capsules across retail pharmacy chains in January 2025. A representative sample of the top six US retail pharmacy chains was selected based on market share and comprehensive geographic distribution.^26^ To gather quantitative pricing data, relevant Vitamin D3 products were identified, the basic cost per unit was calculated, and standardized to cost per 4000 IU. This common reference point was then averaged across pharmacy chains to establish a reliable figure for the average cost of a standard 30-day supply in the US market, as informed by prior supplementation literature.^27^ The annual cost of vitamin D_3_ supplementation was estimated at $60. This per-person intervention cost reflects the $60 annual supplement cost applied to eligible individuals (BMI ≥25), discounted at 3% annually and adjusted for attrition as individuals progressed to diabetes or died. Although clinical trials necessarily include screening, enrolment, and protocol-driven follow-up activities, these trial-specific components do not reflect the costs incurred when vitamin D supplementation is implemented in routine clinical or public health settings. Screening for prediabetes and provision of basic lifestyle advice are already part of usual care for adults at risk of diabetes, and the 2024 Endocrine Society Guideline recommends empiric vitamin D supplementation without routine 25-hydroxyvitamin D testing.^19^ Nonetheless, in a sensitivity analysis, we increased the annual intervention cost to $72 to account for modest additional implementation costs, including brief clinical counselling time (estimated at $10 per year based on approximately 1.5 min of provider time for single-nutrient supplementation recommendations at current physician wage rates^28^^,^^29^) and patient education materials $2 per year.^19^^,^^28^

**Table 2:** The annual per-person transition probabilities, cost and disutility of the intervention, diabetes and disease-related comorbidities.

Parameter	First-year cost $, (SE)	Following-year cost $, (SE)	Source	Parameter	First-year disutility (SE)	Following-year disutility (SE)	Source
Base cost (Normoglycemia)	2636	Annual age-cost increment (Δ $/year): 12 (0)	22	Baseline QALY (Normoglycemia, prediabetes)	0.935	N/A	24
Base cost (Prediabetes)	3244	Annual age-cost increment (Δ $/year): 12 (0)	22	Duration	−0.008 (0)	N/A	24
Type 2 diabetes (T2D)	11,322 (541)	Annual age-cost increment (Δ $/year): −78 (9)	22	Baseline QALY (Type 2 diabetes)	0.935	N/A	24
Macroalbuminuria	13,995 (383)	3717 (354)	22	Smoking	−0.006 (0.005)	N/A	24
Foot ulcer	13,431 (856)	2611 (832)	22	Foot ulcer	−0.017 (0.009)	−0.020 (0.009)	24
Amputation	30,410 (4581)	0 (0)	22	Amputation	−0.092 (0.028)	−0.150 (0.034)	24
Blindness	15,802 (2107)	2892 (2205)	22	Blindness	−0.045 (0.010)	−0.023 (0.010)	24
CKD stage 4–5: eGFR <30 mL/min/1.73 m^2^	13,995 (383)	3717 (354)	22	CKD stage 4–5: eGFR <30 mL/min/1.73 m^2^	−0.043 (0.010)	−0.025 (0.010)	24
CKD stage 3–5: eGFR <60 mL/min/1.73 m^2^	13,995 (383)	3717 (354)	22	CKD stage 3–5: eGFR <60 mL/min/1.73 m^2^	−0.014 (0.003)	−0.015 (0.003)	24
Dialysis	114,585 (5273)	120,361 (6160)	22	Dialysis	−0.038 (0.015)	−0.015 (0.013)	24
Neuropathy	5257 (195)	2447 (226)	22	Neuropathy	−0.007 (0.004)	−0.007 (0.004)	24
Diabetic retinopathy requiring laser treatment	5342 (1210)	2678 (336)	22	Diabetic retinopathy requiring laser treatment	−0.011 (0.007)	−0.014 (0.006)	24
Myocardial infarction (MI)	55,025 (1463)	10,424 (1346)	22	Myocardial infarction (MI)	−0.027 (0.009)	−0.007 (0.008)	24
Stroke	28,916 (1034)	5750 (1065)	22	Stroke	−0.107 (0.015)	−0.051 (0.014)	24
Congestive heart failure (CHF)	37,942 (1119)	8587 (1074)	22	Congestive heart failure (CHF)	−0.050 (0.014)	−0.043 (0.014)	24
Coronary heart disease requiring revascularization	24,719 (681)	0 (0)	22	Coronary heart disease requiring revascularization	−0.005 (0.006)	0 (0.007)	24
Angina	10,831 (402)	0 (0)	22	Angina	−0.015 (0.009)	−0.028 (0.008)	24
Hypoglycemia (Medical Assistance)	9310 (360)	N/A	22	Hypoglycemia (Medical assistance)	0 (0)	N/A	24
Annual vitamin D supplementation (cholecalciferol)	60 (6)	60 (6)		Hypoglycemia (Any assistance)	0 (0.006)	N/A	24
Annual probability reduction in onset risk	15% (7.65%)^a^		1	BMI (One-unit increase)	−0.003 (0)	N/A	24

Event costs and disutilities with standard errors (SE) in parentheses.

a The variance for intervention risk reduction was selected based on changes significantly different than zero at the 5% level, with these significance levels converted to the corresponding standard deviation for the distribution in the model.

### Utilities

In the context of health economics, utility is a measure representing health-related quality of life, that is the value individuals place on different health states. Disutility, conversely, refers to the reduction in quality of life caused by specific health conditions or complications. Within the model, the patient utility equation for individuals with type 2 diabetes begins with a baseline quality of life (the intercept). From this baseline, reductions in quality of life (utility decrements) are applied when the individual experiences a complication in the current year or has a history of complications in the prior years of the model. Furthermore, prediabetes itself is associated with patient disutility, indicating a negative impact on quality of life.^24^ These specific event-related disutilities and baseline values are entered into the model as described in Table 2 to calculate QALYs.

### Adverse events

We assumed there were no adverse events associated with vitamin D use. Based on results of a systematic review,^2^ there was no observed difference in the rate ratios for adverse events such as kidney stones, hypercalcemia, hypercalciuria, or death between those who used and did not use vitamin D. Thus, we did not include any adverse events additional to those described in Table 2 in our simulation.

### Statistical analysis

All cost-effectiveness outcomes were estimated as the mean of 100 runs of 10,000 simulated individuals. Uncertainty is reported as the 2.5th–97.5th empirical percentile (95% uncertainty interval) of the across-runs distribution for each outcome. Simulation runs were performed using the CDC/RTI Prediabetes Microsimulation Model; post-processing of cost-table outputs to compute empirical 95% uncertainty intervals across the model runs were performed in Python (version 3.11; pandas 2.3); visualisations of sensitivity and scenario analyses were produced in R version 4.1.2 (http://www.r-project.org).

#### Base case analyses

All primary results are derived from a deterministic base case analysis applied to the input parameters described in Table 2. A lifetime horizon was selected for the core analysis, simulating an individual until he or she died, to capture the entire health and cost consequences of vitamin D supplementation. The microsimulation thus follows each individual until death using mortality equations validated against cohort studies, as detailed in Hoerger et al.^21^ For each outcome, 95% uncertainty intervals (UI) were calculated as the 2.5th and 97.5th percentiles of the distribution across model runs.

#### Subgroup analyses

We conducted several subgroup analyses of the base case across the lifetime, including age, body weight status (BMI) categories, glycaemic control (based on baseline FPG levels), and family diabetes history. These analyses examined if the intervention was more cost-effective in groups expected to benefit more from the intervention due to greater baseline disease risk.

#### Scenario analyses

We also conducted several scenario analyses. The variability of results from the primary lifetime analysis was assessed to investigate whether intervention in low-risk groups may not be as cost-effective as in high-risk groups. For our base-case analysis, we applied the intervention over the patient’s lifetime with a consistent annual relative reduction in risk across waves.^2^ Thus, we examined the cost-effectiveness of vitamin D supplementation with a 10-year analytical horizon. These results could be used by entities with a shorter budget planning horizon. To be more conservative, we further modelled a scenario whereby the intervention was only provided for 3 years. Additionally, we evaluated a particularly pessimistic scenario wherein risk reduction from supplementation was not consistent, declining to zero after 5 years, while intervention costs continued until individuals receiving the intervention progressed to diabetes or died. We similarly repeated our base-case analysis modelling a lowered WTP threshold of $50,000 per QALY.

#### One-way sensitivity analyses

We conducted a one-way sensitivity analysis to investigate structural uncertainties, adjusting the relative risk reduction and cost of intervention to be 50% higher and lower than the baseline to address parameter uncertainty. In health economics, future medical cost-savings and quality of life are often discounted to reflect the preference of society for benefits received now versus the future, consequently, we also varied the discount rate for costs and QALYs from 0 to 5% to reflect variations in societal preferences.

#### Probabilistic sensitivity analysis

In a probabilistic sensitivity analysis (PSA), we drew model parameters for risk factors, intervention effects, costs, patient disutilities, and complication and mortality equations randomly from appropriate distributions across multiple iterations. The point estimates and the standard errors for the PSA are described in Table 2 and were converted into the corresponding parameters for these distributions in the model, varying multiple model parameters simultaneously to incorporate the impact of all parameter uncertainty on our results. The number of times a new set of model parameters was drawn from the distributions for each variable was set to 100 iterations of 10,000 individuals. We plotted the results of these multiple iterations of the model in a cost-effectiveness scatterplot to visualise the degree of variability in the incremental costs, QALYs, and ICERs of our base lifetime scenario, delineated across quadrants of cost-effectiveness (cost-saving, cost-effective/not cost-effective, more expensive, and less effective).

### Ethics statement

This study used publicly available, de-identified data from NHANES and simulated cohort data from a validated microsimulation model. No individual participant data were collected. The NHANES protocol was approved by the National Center for Health Statistics Research Ethics Review Board, and all NHANES participants provided written informed consent. No additional institutional review board approval was required for this modelling study.

### Role of the funding source

Research Ireland was the funding source for the study and had no role in the study’s design, conduct, or reporting or in the decision to submit the paper for publication. Federal government employee authors conducted the work as a part of their official duties.

## Results

The lifetime cumulative incidence of diabetes fell by 8% (95% UI 6.4–10.6%) relative to the non-intervention group (Fig. 1; Table 3). This led to a reduction in the mean cumulative incidence of diabetes-related complications, with the smallest decline seen for angina (1.8%) and the largest declines seen in diabetic retinopathy requiring laser treatment (9.20%), hypoglycemia (8.96%), and foot ulcer (8.79%) (95% UIs for individual complications are given in Table 3). The number of QALYs increased by 0.12 (95% UI 0.05–0.19; intervention arm 16.47 versus no-intervention arm 16.35). By delaying or avoiding the onset of diabetes, the intervention reduced the cost of diabetes complications and treatment enough to generate a net cost saving of $3208 per person (95% UI cost saving of $5974 to a small added cost of $171). Thus, compared with no supplementation, the provision of vitamin D increased QALYs and lowered medical costs, indicating the intervention was cost saving from a health system perspective. These results held in the shorter 10-year analytical time horizon. Compared to no vitamin D, supplementation was estimated to reduce the 10-year cumulative incidence of diabetes by 10% (95% UI 7.1–12.8%) (Table 3). Because diabetes risk has accumulated for a shorter period, the denominator (total potential cases) is smaller at 10 years versus lifetime, such that the same benefit results in a slightly larger percentage reduction for the 10-year horizon—even though the absolute number of diabetes cases averted for the lifetime horizon is ultimately greater, as described in Table 3. The analysis demonstrates that over this time horizon, declines in complications range from approximately 1%–12%, with the largest reductions seen for hypoglycemia (12%) and foot ulcers (11%) and the smallest declines for myocardial infarction (0.77%), eGFR levels <30 mL/min/1.73 m^2^ (1.33%), and coronary heart disease requiring revascularization (1.41%), generating cost savings of $401 (95% UI −$908 to $192) per person.

**Fig. 1:**
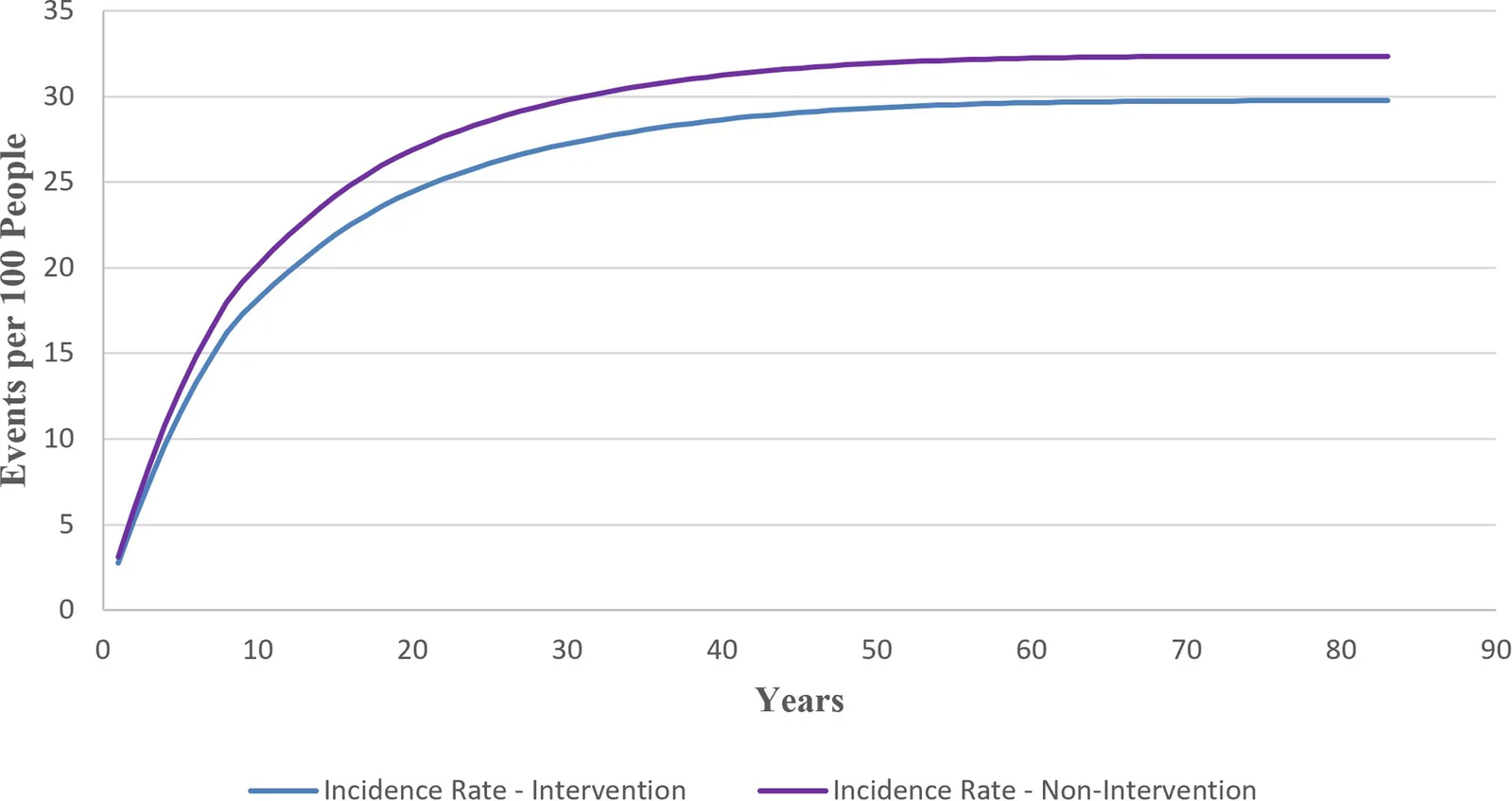
Cumulative Diabetes Incidence. Cumulative incidence of type 2 diabetes in the simulated prediabetes cohort over the lifetime horizon, comparing the vitamin D supplementation arm with the non-supplementation arm. Curves represent the mean of 100 runs of 10,000 individuals, plotted year-by-year. The year-83 endpoints (29.75 intervention; 32.35 no-intervention) correspond to the lifetime row of Table 3; the relative reduction at the lifetime endpoint is 8.04% (95% UI 6.4–10.6%).

**Table 3:** Remaining life-years, costs, QALYs, and cumulative complications for intervention and non-intervention simulations.

Outcome	10-Years			Lifetime		
	Non-Intervention	Intervention	Incremental Effects	Non-Intervention	Intervention	Incremental Effects
Per person						
Remaining life-years (undiscounted)	9.750 (95% UI 9.723–9.774)	9.751 (95% UI 9.722–9.778)	0.001 (95% UI −0.018 to 0.018)	36.45 (95% UI 36.12–36.75)	36.72 (95% UI 36.45–37.09)	0.27 (95% UI 0.06–0.50)
Costs (discounted)	$50,709 (95% UI $50,097 to $51,470)	$50,308 (95% UI $49,682 to $51,138)	−$401 (95% UI −$908 to $192)	$163,210 (95% UI $160,148 to $166,666)	$160,002 (95% UI $156,707 to $163,582)	−$3208 (95% UI −$5974 to $171)
QALYs (discounted)	6.90 (95% UI 6.88–6.92)	6.91 (95% UI 6.89–6.92)	0.01 (95% UI −0.01 to 0.02)	16.35 (95% UI 16.23–16.44)	16.47 (95% UI 16.38–16.59)	0.12 (95% UI 0.05–0.19)
Incremental cost-effectiveness ratio (ICER) ($/QALY)			−$61,122 (95% UI −$1,033,808 to $1,005,279)			−$26,134 (95% UI −$72,486 to $3947)
Net Monetary Benefit (NMB)			$1058 (95% UI −$172 to $1964)			$15,483 (95% UI $8108 to $21,907)
Treatment costs						
Treatment Costs	$34,903 (95% UI $34,704 to $35,191)	$34,420 (95% UI $34,195 to $34,684)	−$483 (95% UI −$608 to −$289)	$93,239 (95% UI $92,437 to $94,127)	$91,975 (95% UI $91,010 to $92,776)	−$1264 (95% UI −$1819 to −$620)
Complication costs						
Complication costs	$15,806 (95% UI $15,297 to $16,541)	$15,532 (95% UI $15,001 to $16,216)	−$274 (95% UI −$725 to $285)	$69,971 (95% UI $67,475 to $73,477)	$67,284 (95% UI $64,533 to $69,805)	−$2687 (95% UI −$5138 to $162)
Intervention costs						
Intervention costs	$0 (95% UI $0 to $0)	$356 (95% UI $352 to $361)	$356 (95% UI $352 to $361)	$0 (95% UI $0 to $0)	$742 (95% UI $731 to $753)	$742 (95% UI $731 to $753)
Mean cumulative incidence rate (events per 100 people)						
Diabetes	20.14 (95% UI 19.46–20.83)	18.15 (95% UI 17.54–18.91)	−9.89% (95% UI −12.75 to −7.14)	32.35 (95% UI 31.53–33.13)	29.75 (95% UI 28.93–30.58)	−8.04% (95% UI −10.57 to −6.37)
Disease complications:						
Amputation	0.05 (95% UI 0.01–0.10)	0.05 (95% UI 0.01–0.11)	−6.11% (wide; rare events)	2.44 (95% UI 2.15–2.82)	2.26 (95% UI 1.99–2.59)	−7.35% (95% UI −19.61 to 6.82)
Angina	2.47 (95% UI 2.12–2.81)	2.43 (95% UI 2.13–2.73)	−1.57% (95% UI −9.88 to 7.72)	11.38 (95% UI 10.81–11.98)	11.17 (95% UI 10.49–11.88)	−1.81% (95% UI −7.14 to 3.11)
Blindness	0.92 (95% UI 0.76–1.09)	0.82 (95% UI 0.66–1.03)	−10.42% (95% UI −30.35 to 14.94)	9.72 (95% UI 9.25–10.29)	8.92 (95% UI 8.29–9.51)	−8.31% (95% UI −13.23 to −2.15)
Congestive heart failure (CHF)	0.98 (95% UI 0.79–1.15)	0.94 (95% UI 0.76–1.12)	−3.8% (95% UI −22.06 to 18.53)	24.70 (95% UI 23.83–25.81)	23.27 (95% UI 22.32–24.61)	−5.76% (95% UI −9.33 to −2.61)
Death	6.57 (95% UI 5.98–7.23)	6.57 (95% UI 6.03–7.19)		100.00 (95% UI 100.00–100.00)	100.00 (95% UI 100.00–100.00)	
Dialysis	0.16 (95% UI 0.08–0.27)	0.15 (95% UI 0.08–0.23)	−5.16% (wide; rare events)	4.55 (95% UI 4.14–4.94)	4.17 (95% UI 3.79–4.60)	−8.43% (95% UI −17.98 to 0.58)
CKD stage 4–5: eGFR <30 mL/min/1.73 m^2^	1.63 (95% UI 1.35–1.85)	1.61 (95% UI 1.34–1.81)	−1.33% (95% UI −18.80 to 19.21)	27.26 (95% UI 26.40–28.19)	26.33 (95% UI 25.46–27.26)	−3.41% (95% UI −6.67–0.45)
CKD stage 3–5: eGFR <60 mL/min/1.73 m^2^	14.69 (95% UI 14.03–15.43)	14.47 (95% UI 13.67–15.21)	−1.46% (95% UI −5.15 to 3.91)	55.02 (95% UI 53.92–56.29)	53.95 (95% UI 52.73–55.47)	−1.95% (95% UI −3.38 to −0.48)
Hypoglycemia (Any assistance)	0.45 (95% UI 0.32–0.58)	0.40 (95% UI 0.28–0.51)	−12.21% (95% UI −38.96 to 33.80)	5.77 (95% UI 5.19–6.43)	5.25 (95% UI 4.72–5.70)	−8.96% (95% UI −21.32 to 3.53)
Hypoglycemia (Medical assistance)	1.07 (95% UI 0.90–1.27)	0.96 (95% UI 0.76–1.14)	−10.43% (95% UI −30.66 to 9.62)	10.89 (95% UI 10.05–11.64)	10.00 (95% UI 9.31–10.95)	−8.18% (95% UI −16.38 to 0.18)
Diabetic retinopathy requiring laser treatment	1.05 (95% UI 0.85–1.29)	0.95 (95% UI 0.77–1.11)	−9.64% (95% UI −25.20 to 16.77)	7.49 (95% UI 7.00–8.13)	6.80 (95% UI 6.28–7.31)	−9.20% (95% UI −16.07 to −1.49)
Macroalbuminuria	0.84 (95% UI 0.65–1.01)	0.75 (95% UI 0.62–0.90)	−10.07% (95% UI −29.72 to 18.15)	15.80 (95% UI 14.96–16.62)	14.45 (95% UI 13.58–15.51)	−8.56% (95% UI −12.30 to −3.83)
Myocardial infarction (MI)	2.92 (95% UI 2.61–3.26)	2.90 (95% UI 2.61–3.31)	−0.77% (95% UI −7.87 to 10.13)	16.40 (95% UI 15.44–17.43)	15.78 (95% UI 14.72–16.82)	−3.75% (95% UI −8.77 to 2.83)
Microalbuminuria	4.59 (95% UI 4.25–4.95)	4.11 (95% UI 3.68–4.45)	−10.48% (95% UI −17.88 to −2.38)	24.97 (95% UI 24.03–25.89)	22.93 (95% UI 22.11–23.87)	−8.2% (95% UI −10.91 to −5.59)
Neuropathy	11.01 (95% UI 10.47–11.55)	9.96 (95% UI 9.43–10.43)	−9.47% (95% UI −13.22 to −5.05)	30.60 (95% UI 29.82–31.37)	28.15 (95% UI 27.34–29.02)	−8.02% (95% UI −10.72 to −5.89)
Coronary heart disease requiring revascularization	5.61 (95% UI 5.22–6.08)	5.54 (95% UI 5.09–5.98)	−1.41% (95% UI −8.94 to 6.51)	29.38 (95% UI 28.01–30.80)	28.49 (95% UI 27.00–29.94)	−3.04% (95% UI −6.93 to 1.97)
Stroke	1.60 (95% UI 1.35–1.87)	1.58 (95% UI 1.33–1.86)	−1.62% (95% UI −13.42 to 12.74)	15.16 (95% UI 14.16–16.29)	14.46 (95% UI 13.32–15.80)	−4.59% (95% UI −10.48 to 2.23)
Foot ulcer	1.08 (95% UI 0.86–1.31)	0.96 (95% UI 0.77–1.19)	−11.02% (95% UI −30.29 to 17.37)	9.70 (95% UI 9.16–10.33)	8.85 (95% UI 8.25–9.58)	−8.79% (95% UI −16.54 to 0.34)

Results are for both full analytical time horizons: lifetime and 10-year. Numbers in parentheses (italicised) are 95% uncertainty intervals (UIs), calculated as the 2.5th and 97.5th empirical percentiles of the distribution of 100 analysis runs (simulated cohort of 10,000 individuals). For relative-reduction columns, UIs are reported with the same sign convention as the point estimate: negative values denote a reduction in incidence with intervention and positive values denote an increase. Some rare-event complications (amputation, dialysis at 10 years) have very wide UIs because a small absolute number of events makes per-run relative reductions volatile; the lifetime horizon is more interpretable for those outcomes. Data are presented as means or totals. Costs and QALYs are discounted back to the present year. Remaining life-years are reported without discounting to reflect that life expectancy is commonly measured in years, not discounted years. The cumulative incidence is shown for the total population in the simulation. The incremental cost-effectiveness ratio (ICER) point estimate is reported as the ratio of mean incremental cost to mean incremental QALYs; the 95% UI on the ICER row is the 2.5/97.5 empirical percentile of the per-run ICER distribution, which is very wide for both horizons because individual runs with incremental QALYs close to zero produce extreme per-run ICERs. For a single-number summary of uncertainty around cost-effectiveness, the net monetary benefit (NMB) at the willingness-to-pay [WTP] threshold of $100,000/QALY is more reliable because it is well-defined under every parameter draw. While negative ICER values demonstrate the intervention as cost-saving across time horizons, the absolute values of negative ICERs cannot be used to compare the degree of cost-effectiveness; NMBs are reported instead, whereby a higher NMB indicates greater cost-effectiveness.

### Subgroup analysis

The cost-effectiveness of vitamin D supplementation increased as BMI increased, with the most significant benefits found in individuals with a BMI ≥40 (class 3 obesity), who had the highest NMB of $25,260 (Table 4). Similarly, intervention in individuals aged <65 years is associated with higher NMB ($19,289) compared to those aged ≥65 years ($15,350). When we restricted our analysis to only those individuals with a family history of diabetes, we observed a higher NMB ($18,590) compared to studying those with no family history of the condition ($14,466). Consistent benefits were observed across levels of glycaemic control, with more substantial economic benefits from vitamin D supplementation realised in subgroups with higher FPG (≥106 mg/dL) rather than lower FPG (<106 mg/dL), accruing NMBs of $19,270 and $9,113, respectively. Overall, intervention consistently provided benefits across all subgroups.

**Table 4:** Subgroup and scenario analysis results.

	ICER ($/QALY)	NMB ($)
Core analysis	−$26,134	$15,483
Subgroup analysis:		
Baseline BMI < 25^a^	−$17,064.17	$9447
BMI 25–30	−$23,170.46	$14,018
BMI < 30	−$19,425.05	$11,919
Obesity (all classes): BMI ≥ 30	−$29,888.59	$20,618
Obesity (Class 1–2): BMI 30–40	−$29,635.11	$19,190
Obesity (Class 3): BMI ≥40	−$35,717.53	$25,260
Age < 65	−$27,844.26	$19,289
Age ≥ 65	−$23,451.53	$15,350
FPG < 106	−$19,329.56	$9113
FPG > 106	−$28,397.82	$19,270
No history	−$19,436.88	$14,466
100% family history	−$33,692.28	$18,590
Scenario analysis:		
Intervention provided for 3 years only	−$33,774.33	$3774
Declining efficacy over 5 years—costs for duration of prediabetes	−$26,963.70	$3438
Higher implementation costs	−$30,082.08	$15,872
Willingness to pay threshold decreased 50%	−$26,134.49	$9346
Different model starting seed	−$28,043.77	$16,515

Data are presented as incremental cost-effectiveness ratios (ICERs) and net monetary benefits (NMBs). Negative ICER values are reported to demonstrate cost savings per QALY gained; however, such values are non-interpretable when comparing levels of cost-effectiveness across interventions. Thus, NMBs are reported, whereby greater positive values indicate greater levels of cost-effectiveness between subgroups and scenarios. All NMBs are calculated based on a WTP of 100,000/QALY, aside from the scenario analysis that considers the effects of decreasing this threshold by 50%. FPG describes fasting plasma glucose.

a The ‘Baseline BMI < 25’ row was generated by a separate full re-run of the simulation in which the cohort was drawn from the NHANES data to represent US adults with prediabetes whose baseline BMI is <25, simulated over a lifetime horizon. Because the CDC/RTI model is a discrete-time microsimulation with annual steps in which risk factors, including BMI, evolve year-on-year per the model’s trajectory equations, individuals whose baseline BMI is <25 kg/m^2^ may cross the BMI ≥ 25 kg/m^2^ supplementation threshold during follow-up. ICER and NMB values thus represent their exposure.

### Scenario analysis

Our findings remained robust to alternative scenarios, with a consistently positive NMB described in Table 4. Scenarios of a shorter analytical horizon had the biggest influence, decreasing cost-effectiveness by 76% when the intervention was provided for 3 years only or 78% when efficacy was modelled as declining over 5 years. In addition, changing assumptions about willingness to pay thresholds and compliance rates decreased cost-effectiveness by 40% and 47%, respectively. Regardless, every scenario remained cost-saving. To address potential implementation costs beyond the supplement itself, we conducted an additional analysis increasing the annual intervention cost from $60 to $72 per person to account for brief clinical counselling and patient education materials. Under this more conservative assumption, the intervention remained cost-saving, with a NMB of $15,872—comparable to the base case findings.

### One-way sensitivity analysis

As described in Fig. 2, across the univariate analyses, the most significant influence on the base-case NMB came from treatment efficacy and discount rates. Cost-effectiveness decreased by 48% with a 50% lower relative risk reduction from supplementation, while changing discount rates to 5% decreased this by 42%. Within a relatively narrow range, there was a limited influence of variation in cost, with cost-effectiveness falling by 2% when intervention costs increased by 50%. Nonetheless, the intervention was still cost-saving in all these scenarios. This finding suggests that our results would remain robust even if implementation costs beyond the supplement itself—such as brief clinical contact or patient education—were also added to our model.

**Fig. 2:**
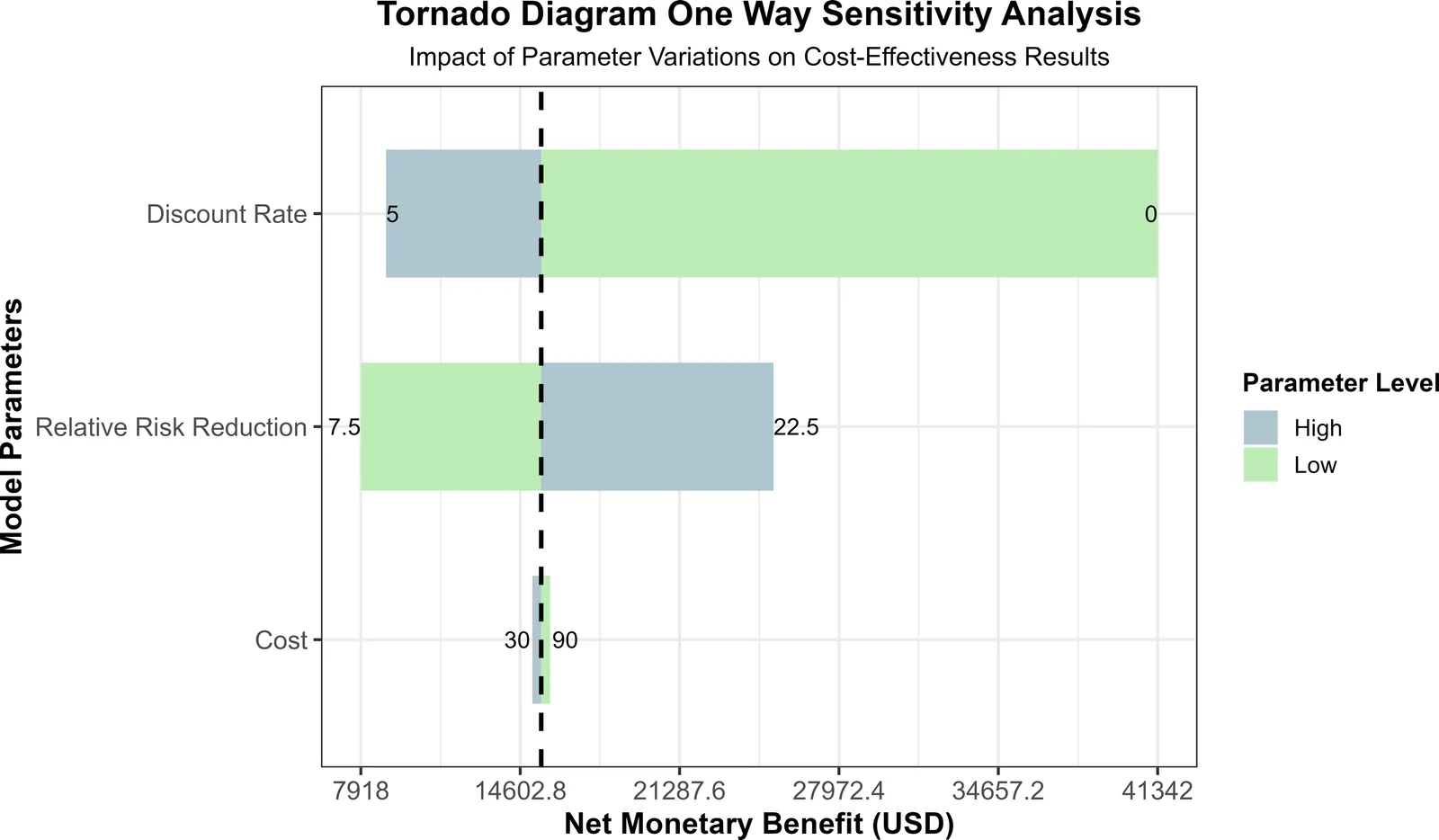
Tornado Diagram One Way Sensitivity Analysis Results. Figure denotes parameter levels as high or low to correspond to parameter values above or below the median point estimates reported in Table 2, evaluating intervention costs at ±50%, relative risk reductions at ±50%, and discount rates at 0% and 5%. This highlights where differences in the magnitude of input values result in changes in the expected outcome value to illustrate which parameters drive the majority of the variation in cost-effectiveness. The dashed line represents the base case value.

### Probabilistic sensitivity analysis

We visualised the results of the PSA of the core lifetime scenario per Fig. A1 in the Appendix, where 99% of ICER values are cost-saving, all are cost-effective, and the mean of the PSA ICER values are consistent with the base case deterministic cost-effectiveness point estimate.

## Discussion

We found that vitamin D supplementation could be a cost-saving strategy for the purpose of preventing type 2 diabetes in adults with prediabetes in the United States. This conclusion is robust with changes in many key model parameters associated with adherence, effectiveness, sustainability, and cost of the intervention and under many unfavourable intervention scenarios. These findings indicate that supplementation of vitamin D is superior to no supplementation. The favourable cost-effectiveness profile of vitamin D supplementation arises from several converging factors. First, the high cumulative incidence of diabetes among individuals with prediabetes indicates that even a modest intervention effectiveness of about 15% has significant long-term effects on the number of cases and the duration that individuals live without diabetes. Second, the supplement’s low cost and minimal need for intervention-related health care contact significantly offset the substantial reduction in treatment costs and health care utilization linked to later diabetes complications for those who would have otherwise developed diabetes sooner. Third, QALYs increase due to the downstream reductions in diabetes-related complications that significantly impact health-related quality of life related to diabetes complications such as heart disease, kidney disease, diabetic eye disease, and the risk of foot ulcers or amputations.

Cost-effectiveness analyses of type 2 diabetes prevention have shown that intensive, multi-disciplinary lifestyle interventions are generally cost-effective, mainly in persons at the highest end of the prediabetes risk spectrum.^3^^,^^26^ We are unaware of any prior research examining the cost-effectiveness of vitamin D supplementation for diabetes prevention, and the Endocrine Society Guideline on Vitamin D for the Prevention of Disease explicitly identified the evaluation of cost-effectiveness analyses as a critical area for future inquiry.^19^ Our current study addresses this knowledge gap, providing insights into the economic viability of vitamin D supplementation in the context of diabetes prevention.

While the reduction in diabetes risk observed in clinical trials with vitamin D is smaller than that achieved through intensive lifestyle interventions or metformin, vitamin D offers distinct benefits. Even a modest reduction at the individual level may be expected to translate into a substantial public health impact where the intervention is particularly tolerable. Our model estimates that vitamin D supplementation reduces the lifetime incidence of diabetes by 2.60%. Assuming there are approximately 98 million U.S. adults with prediabetes,^1^ if each of these adults received vitamin D supplementation, we would expect an estimated 2.5 million fewer cases of diabetes over their lifetimes. Importantly, unlike other interventions, such as intensive lifestyle changes or metformin, which can be burdensome, costly ($570/year for group-based program to $8000/year for DPP-like program; $204/year for metformin), pose significant risks, or have limited scalability, vitamin D is affordable, safe, and feasible for widespread use. In the vitamin D trials and in our model, empirical vitamin D was used without the need to test for blood 25(OH)D levels (the principal vitamin D circulating metabolite), thereby decreasing its cost and aligning with the Endocrine Society Guideline.^19^ These features position vitamin D as a tool to help mitigate the progression of type 2 diabetes in high-risk individuals, making a contribution to public health efforts aimed at addressing this escalating challenge. If Vitamin D is integrated into primary care recommendations and broader public health promotion efforts, it may be a practical and impactful strategy for reducing the progression to type 2 diabetes in high-risk individuals. It is important to note that while vitamin D supplementation may be effective and cost-saving in lowering the risk of type 2 diabetes in adults with prediabetes, it should complement—not replace—other strategies, such as lifestyle modifications focused on weight management.

Although the present analysis was based on the U.S. population, the findings are relevant to the much larger global population at risk for diabetes. In 2022, an estimated 828 million adults worldwide were affected by diabetes, an increase of 360 million since 1990.^3^ The largest increases occurred in low- and middle-income countries in Southeast Asia, South Asia, the Middle East, North Africa, and Latin America and the Caribbean.^3^ Notably, nearly 60% of adults over age 30 with diabetes are not receiving treatment, highlighting an urgent need for effective diabetes prevention strategies, especially those that are low-cost and accessible.

In this context, vitamin D supplementation has several attributes that support its feasibility as a public health intervention in resource-limited settings. Vitamin D is inexpensive, widely available globally (often as an over-the-counter supplement or through existing pharmaceutical supply chains), and requires minimal clinical infrastructure for delivery. Even if the individual-level effect on diabetes risk is modest, the combination of low cost, ease of administration, and favorable safety profile suggests that vitamin D supplementation could be highly cost-effective when implemented at scale. In contrast, resource-intensive lifestyle interventions and high-cost pharmacologic therapies are unlikely to be broadly deployed in many low-resource settings. Cost-effectiveness analyses are therefore particularly informative, as they help determine whether small individual-level benefits from a low-cost intervention could translate into meaningful population-level health gains. While country-specific analyses would be needed to inform local policy decisions, our findings provide a useful framework for considering vitamin D supplementation as part of global diabetes prevention strategies. Ethnic minorities—particularly non-Hispanic Black individuals—have a higher prevalence of vitamin D deficiency but are less likely to use vitamin D supplements compared to non-Hispanic White individuals.^30^ Additionally, the prevalence of type 2 diabetes is higher among non-Hispanic Black individuals than non-Hispanic White individuals.^3^ Our study found that vitamin D supplementation is a cost-effective strategy for preventing type 2 diabetes. Therefore, promoting vitamin D supplementation among minority populations could not only reduce the overall number of diabetes cases but also help address disparities across ethnic groups.

The usefulness of vitamin D supplementation in lowering the risk of type 2 diabetes in the general population at average risk is uncertain. Recent studies indicate that vitamin D may modestly reduce the risk of diabetes, even among adults with low to moderate baseline risk. Specifically, two large randomized trials—the Vitamin D and Omega-3 Trial (VITAL, United States) and the Finnish Vitamin D Trial (FIND, Finland) were designed primarily for non-diabetes outcomes, enrolled generally healthy adults, and tested vitamin D supplementation at doses higher than the recommended daily allowance for the general population.^31^^,^^32^ In VITAL, 25,871 participants (mean baseline 25[OH]D level of 31 ng/mL) were randomized to 2000 IU/day of vitamin D_3_ or a placebo and followed for a median of 5.3 years; the hazard ratio for incident diabetes was 0.91 (95% CI, 0.76–1.09). In FIND, 2271 participants (mean baseline 25[OH]D level of 30 ng/mL [75 nmol/L]) were randomized to vitamin D_3_ at either 1600 IU/day or 3200 IU/day or placebo; after an average follow-up of 4.2 years, the hazard ratio for incident diabetes was 0.86 (95% CI, 0.58–1.29). Although these studies show directionally consistent reductions in diabetes risk as in adult pre-diabetes populations, the absolute risk reduction would be expected to be smaller given the lower baseline risk.

In subgroup analyses, we found that the cost-effectiveness of vitamin D supplementation increased with higher baseline BMI. At first glance, this result may appear inconsistent with subgroup analyses in the meta-analysis by Pittas et al.,^2^ which suggested a greater relative effect of vitamin D among individuals with lower baseline BMI. However, subgroup findings from individual trials and meta-analyses are inherently uncertain and should be interpreted with caution, as they are hypothesis-generating and not powered to establish effect modification. Accordingly, our cost-effectiveness model applied the overall mean relative risk reduction observed across the full trial populations. Importantly, individuals with prediabetes and higher BMI have a substantially greater baseline risk of progression to diabetes. Under the assumption of a constant relative risk reduction, this translates into larger absolute risk reductions in higher-risk groups, including those with severe obesity, which in turn improves cost-effectiveness. Thus, the greater cost-effectiveness observed at higher BMI in the microsimulation model reflects differences in baseline risk rather than an assumption of greater biological efficacy of vitamin D in this subgroup. Notably, the subgroup findings from Pittas et al. also suggest that individuals with obesity may require higher vitamin D doses to achieve and maintain optimal 25(OH)D concentrations to derive benefit. Consistent with this, in the D2d study, participants who achieved intra-atrial mean 25(OH)D concentrations ≥40 ng/mL had a significantly lower risk of diabetes than those with lower concentrations, across BMI categories.^33^

We also examined a baseline BMI <25 subgroup, generated as a separate full re-run of the model using a nationally representative NHANES sample of US adults with prediabetes and baseline BMI below 25. Baseline BMI is among the strongest predictors of transition to type 2 diabetes, so this group represents the floor of the inherent-risk distribution within the prediabetes population — a stress test of whether cost-effectiveness holds where the expected absolute benefit is smallest. Because the CDC/RTI model is a discrete-time microsimulation, the model evaluates intervention eligibility annually on time-varying BMI and individuals may cross the eligibility threshold during follow-up as their BMI evolves over the lifetime horizon. Vitamin D supplementation remained cost-saving in this subgroup, though by the smallest margin of any BMI tranche considered. This reflects a favorable but modest intervention effect, consistent with the small fraction of person-years actually exposed during follow-up to accrue intervention costs and benefits.

Our analysis has multiple strengths. We employed a robust, validated model to evaluate the effectiveness of an intervention aimed at diabetes prevention. High-quality data from clinical trials specifically designed to test vitamin D for preventing diabetes in adults with prediabetes were utilized. Key assumptions like costs and disease progression rates are transparent, evidence-based, and well-justified. We applied standard metrics (e.g., ICER) to facilitate comparisons across interventions. Estimating the cost-effectiveness of interventions for chronic diseases poses particular challenges, particularly when costs are immediate, but health benefits materialize years later. Conducting long-term clinical trials in such contexts would be prohibitively expensive and unlikely to generate evidence in a timely manner. Simulation models offer a practical alternative, enabling policymakers to make well-informed decisions. The model used in our analysis has been validated against numerous clinical trials and cohort studies, accurately predicting the onset and progression to diabetes and its complications.^21^ Additionally, the model underwent extensive sensitivity and scenario analyses in this study, consistently showing that vitamin D supplementation is generally cost saving. Our study also has limitations. First, it shares the inherent limitations common to all simulation models, including parameter uncertainty and structural uncertainty. Second, like all simulation studies, it requires assumptions to be made. The accuracy of the study results therefore depends on how closely these underlying assumptions reflect real-world conditions. The meta-analysis by Pittas et al.,^2^ with a median follow-up of 3 years of vitamin D use, required us to make assumptions about costs and effectiveness beyond this timeframe. Sensitivity analyses accounting for declining efficacy and increasing costs over time did not alter the results. Finally, although the mean age for adults with prediabetes was post fifty, all trials enrolled adults aged ≥18 years. Therefore, we set 18 years as the lower age limit in the model to remain faithful to the trial populations and avoid arbitrary age cutoffs unsupported by the trial designs. Using the age limit of 18 also enhances the generalizability and applicability of the findings to the full adult population with diabetes encountered in clinical and public health settings. Additionally, the microsimulation was not parameterised with treatment-effect estimates disaggregated by sex or by race/ethnicity, because the published individual-participant-data meta-analysis^2^ did not detect statistically significant heterogeneity of vitamin D treatment effect across these subgroups and thus did not generate stratum-specific hazard ratios to apply. Nonetheless, should future trial evidence demonstrate clinically meaningful effect modification by sex or race/ethnicity, our subgroup cost-effectiveness estimates can be readily updated to reflect them.

In summary, using a validated microsimulation model to evaluate disease progression, costs, and quality of life, we estimated the lifetime cost-effectiveness of vitamin D supplementation for the U.S. prediabetes population. Our analysis indicates that vitamin D supplementation may be a cost-saving and effective strategy for delaying or preventing diabetes. Addressing the diabetes epidemic requires a comprehensive approach. Integrating vitamin D with other proven interventions—such as lifestyle changes, weight management, and pharmacologic therapies—may significantly lower diabetes risk in the population. However, in low-resource settings, vitamin D may represent a particularly advantageous therapy as a singular cost-saving intervention.

## Contributors

JB, PZ, YS, EG, and AGP conceived and designed the study. PZ, YS, and AGP provided study materials and model access. JB, PZ, YS, EG, and AGP acquired the data. JB conducted the statistical analysis. JB, PZ, YS, EG, and AGP analysed and interpreted the data. JB drafted the manuscript. JB, PZ, YS, EG, and AGP critically revised the manuscript for important intellectual content. EG and JB obtained funding. All authors had full access to all the raw data in the study, JB and YS directly verified the data. All authors have read and approved the final version of the manuscript. JB had final responsibility for the decision to submit the manuscript for publication.

## Data sharing statement

**Data sharing:** All data and code generated for this modelling study will be made available. No new individual participant data were collected by the authors; the analytic cohort was derived from publicly available NHANES 2013–2018 microdata and published sources, and the study used the CDC/RTI Prediabetes Microsimulation Model.

**What will be shared:** a deidentified analytic dataset constructed from NHANES variables used to parameterise the simulated prediabetes cohort; a complete data dictionary; all model input tables (event risks, costs, utilities, distributions) including PSA parameterisation and scenario specifications; R and Python scripts for post-processing and figure/table generation; and deidentified aggregate model outputs for base case, subgroups, one-way and probabilistic sensitivity, and scenario analyses. NHANES raw microdata are not redistributed, but scripts to obtain and process them are provided. The microsimulation model used in this study is a development of the CDC/RTI Prediabetes Microsimulation Model. The underlying source code is the property of the CDC and RTI International and is not publicly distributable. The technical user manual and accompanying technical-report appendices describing model architecture, transition equations, and parameterisation are available from RTI International on reasonable request. The configuration files used to generate all results in this study are available from the corresponding author on reasonable request.

The CDC/RTI microsimulation source code is owned by its developers and cannot be shared by the authors; we will share all input files required to reproduce our results within that model.

**Additional documents:** simulation protocol and statistical analysis plan; README with replication instructions.

**When:** with publication; available indefinitely. Pre-publication access is available on reasonable request.

**Where:** public repository (Mendeley Data) with a permanent DOI provided at publication; pre-publication requests via the corresponding author at jonathanbriody@rcsi.ie.

**Access criteria:** all author-deposited materials will be openly accessible without restriction (no proposal or data access agreement required). For independent use of the CDC/RTI model engine, researchers must obtain permission from the model owners; the authors will provide contact details and share the exact input bundles to enable replication once access is granted.

## Declaration of interests

Dr. Pittas has received a research grant from Abiogen S.p.A, awarded to Tufts Medical Center (Boston, MA) to support an investigator-initiated research study. Dr. Pittas has received travel support from Abiogen S.p.A. to attend the 9th International Consensus Conference on Vitamin D. All other authors declare no competing interests.
