# Supplementary material for: The cost-effectiveness of vitamin D supplementation for the prevention of type 2 diabetes in the United States: a microsimulation modelling study
Source: Lancet Reg Health Am. 2026 Jun 27;61:101543. doi: 10.1016/j.lana.2026.101543 (PMC13728357; doi:10.1016/j.lana.2026.101543)
Supplement: Supplementary file 1 [file mmc1.pdf]

Supplementary Material

The Cost-effectiveness of Vitamin D Treatment for the Prevention of Type 2 Diabetes in the United States: A Microsimulation Modelling Study

Manuscript reference: TLRHAMERICAS-D-25-00981 (Revision 3)

Briody J, Pittas A, Zhang P, Shao Y, Gregg E

Contents

Table A1. The Annual Per-Person Medicare Cost of the Intervention, Diabetes and Disease-Related Complications and Comorbidities

Figure A1. Cost-Effectiveness Scatterplot

Figure A2. Cost-Effectiveness Acceptability Curve

Notes

The CHEERS 2022 Checklist is provided as a separate supplementary file (Supplementary File 1 - CHEERS 2022 Checklist.pdf) and is not included in this combined supplementary appendix.

Appendix:

Table A1. The Annual Per-Person Medicare Cost of the Intervention, Diabetes and Disease-Related Complications/Comorbidities

Event	Medicare First-Year Cost $, (SE)	Medicare Following-Year Cost $, (SE)	Sources
**Annual Vitamin D Supplementation (Cholecalciferol)**	60 (6)	60 (6)	Pharmacy Review
**Base Cost (Normoglycemia)**	2,571	Annual age-cost increment (Δ $/year): 12 (0)	23
**Base Cost (Prediabetes)**	3,164	Annual age-cost increment (Δ $/year): 12 (0)	23
**Type 2 Diabetes (T2D)**	11,491 (5)	Annual age-cost increment (Δ $/year): 0 (0)	23
**Macroalbuminuria**	11,357 (21)	2,224 (20)	23
**Foot Ulcer**	12,374 (31)	2,733 (28)	23
**Amputation**	46,987 (133)	8,902 (161)	23
**Blindness**	7,722 (46)	530 (42)	23
**Incremental Cost in Year of Death**	593 (0)	N/A	23
**CKD stage 4–5: eGFR <30 mL/min/1.73 m²**	11,357 (21)	2,224 (20)	23
**CKD stage 3–5: eGFR <60 mL/min/1.73 m²**	11,357 (21)	2,224 (20)	23
**Dialysis**	64,920 (76)	45,806 (82)	23
**Neuropathy**	5,411 (27)	1,033 (24)	23
**Diabetic retinopathy requiring laser treatment**	809 (45)	0 (0)	23
**Myocardial Infarction (MI)**	22,513 (31)	1,901 (30)	23
**Stroke**	14,756 (23)	190 (26)	23
**Congestive Heart Failure (CHF)**	14,797 (24)	3,547 (24)	23
**Coronary heart disease requiring revascularization**	13,403 (25)	683 (37)	23
**Angina**	6,215 (27)	643 (33)	23
**Hypoglycemia (Medical Assistance)**	11,147 (32)	0 (0)	23

Sensitivity Analysis

The scatterplot in Figure A1 visualises the results of the probabilistic sensitivity analysis (PSA) of the core lifetime scenario. The spread of points along both axes is limited, the majority are clustered around similar values in the lower right quadrant, and 95% of the values fall between -$4,678 and -$101,603/QALY. Consistent results indicate that the intervention generally resulted in cost savings, purely dominating no supplementation in 99% of the simulations by providing negative incremental costs and positive incremental QALYs. Vitamin D produces a higher quality of life (QoL) when compared to no supplementation in every simulation, with incremental QALYs ranging from 0.05 to

0.25. On average, across model iterations, the intervention reduced costs by -$3,701 while increasing QALYs by 0.1220; accordingly, most ICER values are negative, suggesting that vitamin D both reduces costs while providing beneficial health outcomes in this population.

Figure A1: Cost-Effectiveness Scatterplot

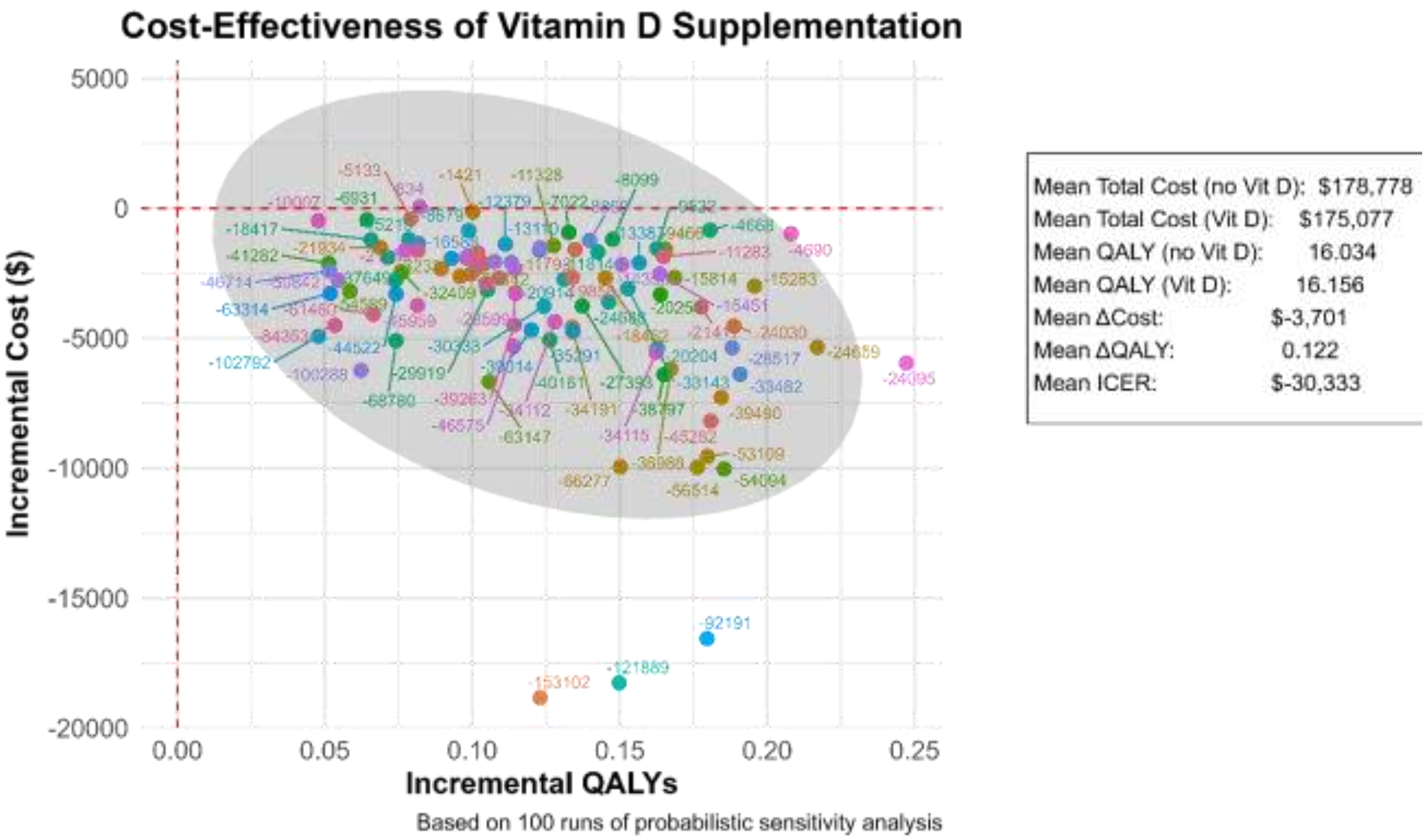

Figure A1 illustrates the additional QALYs gained due to the intervention on the x-axis and the additional costs due to the intervention on the y-axis (positive values indicate higher costs, negative values indicate cost savings). Particularly, the horizontal line at $0 on the y-axis represents the threshold where the intervention costs no more than current care and the vertical line at 0 on the x-axis represents the threshold where the intervention provides no additional QALYs. Each data point represents an individual model run from the 100 repetitions in the probabilistic sensitivity analysis (PSA) where parameter values were randomly drawn from predefined distributions. The associated incremental cost-effectiveness ratio (ICER) values are reported. The ellipse around points represents a 95% confidence incidence.

The cost-effectiveness acceptability curve (CEAC) in Figure A2 assesses the probability that the intervention is cost-effective at different willingness-to-pay (WTP) thresholds per QALY gained, calculated as the proportion of PSA runs where the ICER was less than or equal to each WTP threshold. The results suggest that even if the U.S. health care system was unwilling to pay anything for a QALY, there would still be a 99% probability that supplementation with vitamin D would be cost-effective, due to the cost-saving nature of this intervention. The probability that vitamin D would be preferred to no supplementation reaches 100% with just a small willingness to pay ($5,000/QALY), with this result remaining stable across higher WTP thresholds.

Figure A2: Cost-Effectiveness Acceptability Curve

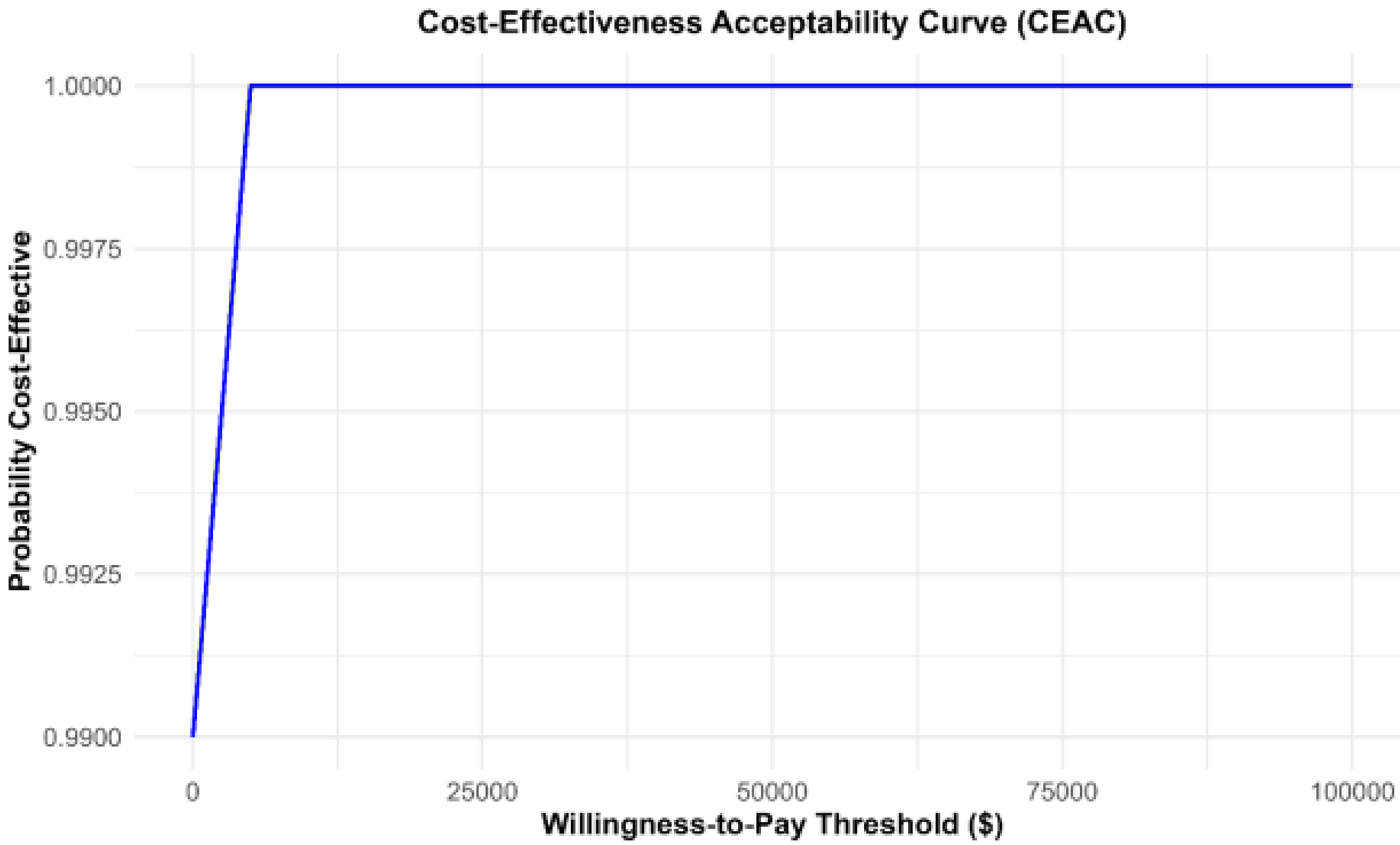

Figure A2 demonstrates WTP thresholds ranging from $0 to $100,000 per QALY on the x-axis. The short range of probabilities of cost-effectiveness on the y-axis, from 99 to 100%, indicates a robust cost-effectiveness profile for vitamin D supplementation, regardless of the WTP threshold applied. The maximum probability is reached at $5,000/QALY, remaining at 100% as thresholds rise.
